# Drug Clearance in Patients with Inflammatory Bowel Disease Treated with Biologics

**DOI:** 10.3390/jcm12227132

**Published:** 2023-11-16

**Authors:** Tina Deyhim, Adam S. Cheifetz, Konstantinos Papamichael

**Affiliations:** Center for Inflammatory Bowel Diseases, Division of Gastroenterology, Beth Israel Deaconess Medical Center, Boston, MA 02215, USA; tdeyhim@bidmc.harvard.edu (T.D.); acheifet@bidmc.harvard.edu (A.S.C.)

**Keywords:** clearance, inflammatory bowel disease, therapeutic drug monitoring, anti-TNF therapy, vedolizumab, ustekinumab, mirikizumab, risankizumab, model informed precision dosing, pharmacokinetic dashboard

## Abstract

Biological therapy is very effective for treating patients with moderate to severe inflammatory bowel disease (IBD). However, up to 40% can have primary non-response, and up to 50% of the patients can experience a loss of response to anti-tumor necrosis factor therapy. These undesirable outcomes can be attributed to either a mechanistic failure or pharmacokinetic (PK) issues characterized by an inadequate drug exposure and a high drug clearance. There are several factors associated with accelerated clearance of biologics including increased body weight, low serum albumin and immunogenicity. Drug clearance has gained a lot of attention recently as cumulative data suggest that there is an association between drug clearance and therapeutic outcomes in patients with IBD. Moreover, clearance is used by model informed precision dosing (MIDP) tools, or PK dashboards, to adjust the dosing for reaching a target drug concentration threshold towards a more personalized application of TDM. However, the role of drug clearance in clinical practice is yet to be determined. This comprehensive review aims to present data regarding the variables affecting the clearance of specific biologics, the association of clearance with therapeutic outcomes and the role of clearance monitoring and MIPD in patients with IBD.

## 1. Introduction

Biological therapy is very effective for treating patients with moderate to severe inflammatory bowel diseases (IBD) such as Crohn’s disease (CD) and ulcerative colitis (UC). However, up to 40% and 80% of the patients can have primary non-response and primary non-remission to anti-tumor necrosis factor (anti-TNF) therapy, respectively [1]. Moreover, up to half of the patients with IBD may experience secondary loss of response [2]. Therapeutic drug monitoring (TDM) can help to explain these unwanted outcomes attributed either to a mechanistic failure in the presence of adequate drug concentration or pharmacokinetic (PK) issues characterized by an inadequate drug exposure and a high drug clearance [3].

**Figure 1:**
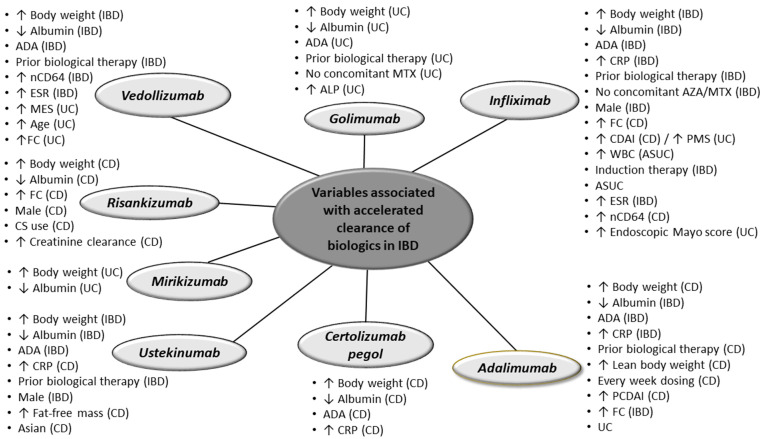
Variables associated with higher clearance of biologics in patients with inflammatory bowel disease. CD: Crohn’s disease; IBD: inflammatory bowel disease; UC: ulcerative colitis; ADA; anti-drug antibodies; CRP: C-reactive protein, AZA: azathioprine, MTX: methotrexate, FC: fecal calprotectin, CDAI: Crohn’s disease activity index, PMS: partial Mayo score, WBC: white blood count, ESR: erythrocyte sedimentation rate, EOW: every other week, PCDAI: pediatric CDAI, ALP: alkaline phosphatase; nCD64: neutrophil CD64; ↑ higher; ↓ lower.

Drug clearance is expressed as the volume of plasma in the vascular compartment that is cleared of drug per unit of time, and it is estimated using Bayesian modeling [3]. Mechanisms underlying the clearance of monoclonal antibodies include intracellular catabolism and endocytosis, increase in target load, protein-losing enteropathy and binding to anti-drug antibodies. There are several factors associated with the above mechanisms that can influence the clearance of biologics including body weight, serum albumin and immunogenicity (Figure 1) [4,5,6,7,8,9,10,11,12,13,14,15,16,17,18,19,20,21,22,23,24,25,26,27,28,29,30,31,32,33,34,35,36,37,38,39,40,41,42,43,44,45,46]. In particular, increased body weight, decreased albumin and anti-drug antibodies (ADAs) have been associated with higher clearance for almost all the biologics currently used in IBD (Figure 1).

Drug clearance has gained a lot of attention recently as an increased estimated baseline clearance may identify patients at high risk of underexposure to biologics and PK-related treatment failure. Furthermore, a sudden increase in clearance during therapy can precede the decrease in drug concentrations and may predict a flare prior to the development of symptoms or low drug concentrations. Moreover, cumulative data suggest that there is an association between drug clearance and therapeutic outcomes in patients with IBD (Table 1) [17,21,22,24,32,36,40,47,48,49,50,51].

Finally, clearance is used by model informed precision dosing (MIDP) tools, or PK dashboards, to adjust the dosing for reaching a target drug concentration threshold, typically depending on the desired therapeutic set by the treating physician. However, the role of drug clearance in clinical practice is yet to be determined.

The aim of this comprehensive review is to present data regarding the variables affecting the clearance of specific biologics, the association of clearance with therapeutic outcomes and the role of clearance monitoring and MIPD in patients with IBD.

## 2. Variables Affecting Clearance of Biologics

### 2.1. Infliximab

Numerous factors have been shown to accelerate infliximab clearance in patients with IBD. The most frequently identified variables from studies were increased body weight, lower albumin and immunogenicity (Figure 1) [4,5,6,7,8,9,10,11,12,13,14,15,16,17,18,19,20]. Other factors that can lead to high clearance include male gender [5,12,18], induction compared to maintenance therapy [14,21,22], prior biologic therapy [13], an increased Crohn’s disease activity index (CDAI) [11], as well as elevated fecal calprotectin [10,11], C-reactive protein (CRP) [14,23,24] and erythrocyte sedimentation rate (ESR) [10]. On the other hand, concomitant immunomodulators (IMMs) (thiopurines/methotrexate) were found to decrease infliximab clearance by around 15% [6,9,17]. This is probably due to the fact that IMM can prevent or suppress immunogenicity as well as decrease the TNF target-antigen burden and consequently the target-mediated elimination of infliximab; although, the mechanisms by which IMM decrease infliximab clearance have not been clearly defined yet [17].

A post hoc analysis of the REACH and ACCENT I randomized controlled trials (RCTs) showed that the clearance was higher in those who developed ADAs or had low baseline albumin [6]. Moreover, concurrent IMM use decreased infliximab clearance by 14% [6]. Data from the Phase I study of maintenance subcutaneous therapy with the infliximab biosimilar CT-P13 in patients with IBD showed that clearance could be increased by 43.2%, 30.1% and 39% by an elevated body weight (from 70 to 120 kg), lower albumin (from 44 to 32 gr/L) and positive ADAs, respectively [13]. The same study showed that patients previously treated with biologics also exhibited a higher drug clearance compared to anti-TNF naïve patients, probably reflecting a more refractory disease and a higher tendency to develop ADAs [13]. A multi-center prospective study including pediatric patients with CD showed that median clearance was higher in ADA-positive compared to ADA-negative patients (0.0111 L/h vs. 0.0094 L/h, *p* < 0.001) [8]. A post hoc analysis of the TAILORIX RCT showed that higher infliximab clearance in patients with CD was associated with increased fecal calprotectin, decreased albumin, increased CDAI and immunogenicity [11]. In a PK analysis of the ACT1 and ACT2, RCTs increased body weight, and lower albumin predicted a higher infliximab clearance in UC. The same study also showed that the mean clearance was 47.1% higher for patients with positive ADAs [12]. A prospective PK study regarding patients with IBD showed that concomitant azathioprine use led to a 15.1% decrease in infliximab clearance [17].

### 2.2. Adalimumab

Several factors have been shown to accelerate adalimumab clearance in patients with IBD including body weight, lower albumin and immunogenicity. (Figure 1) [26,27,28,29,30]. Other factors associated with increased clearance include prior biologic exposure [50], every week dosing [27], UC [29], elevated CRP [29,30] and higher fecal calprotectin [29].

A post hoc analysis of the SERENE CD and UC RCTs identified increased body weight, lower baseline albumin and immunogenicity as variables associated with higher drug clearance [29]. Other factors associated with accelerated adalimumab clearance were UC and elevated baseline fecal calprotectin and CRP levels. The PK analysis of the IMAgINE-1 RCT showed that increased body weight, higher baseline CRP and lower baseline albumin levels were associated with a greater clearance of adalimumab in pediatric CD [30]. In a prospective multicenter study, positive ADAs increased the clearance of a typical patient with CD from 0.330 L/d to 0.525 L/d [26]. Similarly, another study showed a four-fold increase in adalimumab clearance in the presence of ADAs in patients with CD [27].

### 2.3. Certolizumab Pegol

Variables associated with a higher clearance of certolizumab pegol in patients with CD include increased body weight, lower albumin, immunogenicity and elevated CRP (Figure 1) [31,32].

In a certolizumab pegol PK modeling study, body weight (46.8–100.5 kg) increased the median clearance from 82 to 120%; albumin (32–48 g/L) decreased drug clearance from 123 to 85%; CRP (0.5–54.0 mg/L) increased the median clearance from 83 to 113%; and positive ADAs increased the median clearance by 142–174% [31]. A PK analysis on phase 2 and 3 certolizumab pegol clinical trials demonstrated that the predicted baseline certolizumab pegol clearance of ≥0.5 L/d was associated with a higher probability of a sub-therapeutic drug concentration at week 6 [32]. The same study using the PRECiSE 1 and 2 RCTs datasets identified a baseline certolizumab pegol clearance associated with composite remission (CDAI ≤ 150 and fecal calprotectin concentration ≤ 250 μg/g) at both week 6 [odds ratio (OR): 0.92; 95% confidence interval (CI): 0.87–0.96] and week 26 (OR: 0.93; 95%CI 0.88–0.97) [32].

### 2.4. Golimumab

Limited data show that the factors associated with a higher golimumab clearance in patients with UC include increased body weight, lower albumin, immunogenicity, previous biological therapy and a lack of concomitant methotrexate use (Figure 1) [33,34].

A population PK model developed on pooled data from studies regarding adults (NCT00487539 and NCT00488631) and children with moderate-to-severe UC (NCT 01900574), as well as children with polyarticular juvenile idiopathic arthritis (NCT01230827), showed that golimumab clearance increased with body weight and immunogenicity, while the clearance decreased with higher baseline albumin and concomitant methotrexate. Patients receiving methotrexate had a 17% lower clearance compared to those on golimumab monotherapy, and positive ADAs were associated with a 21% higher drug clearance [34]. In the same line, in a PK study of 56 patients with moderate-to-severe UC, golimumab clearance was 31% higher when ADAs were detected [33].

### 2.5. Vedolizumab

Several factors have been identified to accelerate vedolizumab clearance in patients with IBD; the most important ones being increased body weight, lower albumin and immunogenicity (Figure 1) [35,36,37,38,39,40]. Other factors that can lead to a high clearance include prior biological therapy [36,38,39], older age [36], and higher endoscopic Mayo score [35], as well as elevated fecal calprotectin [36,39], CRP [39] and ESR [40].

The prospective multicenter LOVE-CD (NCT02646683) study showed that vedolizumab clearance was higher in patients with CD with lower serum albumin concentrations (+26%, from 41 g/L to 28 g/L), presence of ADAs (+89% compared to no ADAs) and previous exposure to other biologic therapies (+25% compared to no biologic naïve patients) [38]. A PK study of the GEMINI 1 RCT demonstrated that the vedolizumab clearance was higher in patients with UC with a history of prior anti-TNF treatment, lower serum albumin and higher fecal calprotectin [36].

### 2.6. Ustekinumab

Several factors have been identified to accelerate the ustekinumab clearance in patients with IBD including increased body weight, lower albumin and immunogenicity (Figure 1) [41,42,43,44]. Other factors that can lead to a high clearance include prior exposure to biologics [42,43], increasing fat-free mass [43], male gender [42,44], Asian race [42] and higher CRP levels [42].

Data from four phase IIb/III ustekinumab clinical trials (C0743T26, CNTO1275CRD3001, CNTO1275CRD3002, CNTO1275CRD3003) demonstrated that increased body weight, elevated CRP, decreased albumin, TNF antagonist failure status (11% higher in failed patients), immunogenicity (increased by 13% in positive ADA status), sex (17% higher in males) and race (14% higher in Asian compared to non-Asian races) were associated with a higher clearance in CD [42]. In a PK analysis of UNIFI (NCT02407236), clearance increased non-linearly with body weight in patients with UC [44]. Moreover, a higher ustekinumab clearance was associated with lower albumin, male sex and immunogenicity [44].

### 2.7. Risankizumab

A PK analysis from a phase I study in healthy participants (NCT05305222) and phase II and III studies in CD (NCT02031276, ADVANCE, MOTIVATE and FORTIFY) identified increased body weight, decreased albumin, increased fecal calprotectin, corticosteroid use, increased creatinine clearance and male gender as variables associated with higher risakinumab clearance (Figure 1) [45]. Neutralizing antibodies and ADAs were not identified as significant covariates for risankizumab clearance [45].

### 2.8. Mirikizumab

A PK analysis of three RCTs (NCT02589665, NCT03518086, NCT03524092), including 1362 patients with UC, identified increased body weight and decreased albumin as factors associated with higher mirikizumab clearance (Figure 1) [46].

## 3. Association of Clearance with Therapeutic Outcomes

There is cumulating evidence suggesting that a higher clearance is associated with sub-therapeutic drug concentrations and unwanted therapeutic outcomes in patients with IBD, while a lower drug clearance is associated with clinical, biomarker, endoscopic and deep remission (Table 1).

Regarding infliximab, a PK analysis of the ACT 1 and two RCTs found a linear relationship between the baseline infliximab clearance and week 8 Mayo endoscopic scores (MES) (*p* < 0.001). Based on a receiver operating characteristic (ROC) curve analysis, a threshold of <0.397 L/d was associated with week 8 MES of ≤ 1 with a sensitivity (SN), specificity (SP), positive predictive value and area under the curve (AUC) of 75%, 48%, 68% and 0.64, respectively [48]. A prospective study of 31 children with IBD showed that patients who achieved deep remission at week 24 had a lower infliximab clearance at week 6 (0.202 L/d vs. 0.269 L/d, *p* = 0.020) and week 12 (0.215 L/d vs. 0.243 L/day, *p* = 0.022) compared to patients not achieving deep remission [52]. In a retrospective study of patients with acute severe UC, the median baseline calculated clearance was higher in patients requiring a colectomy at 6 months than in patients without a colectomy (0.733 vs. 0.569 L/d; *p* = 0.005). An infliximab clearance threshold of 0.627 L/d identified patients who required a colectomy with 80% SN and 82.8% SP (AUC: 0.80) [47]. A multivariable analysis identified the baseline infliximab clearance as the only factor associated with colectomy [47]. A retrospective study of 36 patients with corticosteroid-refractory acute UC showed that the infliximab clearance increased over time in those requiring a colectomy [14]. A prospective study by Peticollin et al. aiming to explore the link between PK parameters and the probability of relapse after de-escalation of infliximab therapy in patients with IBD showed that a drug clearance higher than 0.320 L/d at the time of infliximab de-escalation was associated with a shorter time to relapse [17].

There are only limited data regarding the association of adalimumab clearance with clinical outcomes in patients with IBD (Table 1). A recent retrospective cohort study including patients with CD showed that the median clearance was lower in patients achieving endoscopic remission as compared to those with persistent active endoscopic disease (0.247 L/d vs. 0.326 L/d, *p* < 0.01) [50]. Of note, there was no significant difference in the median adalimumab concentration between patients with endoscopic remission compared to those without (9.3 μg/mL vs. 11.7 μg/mL), implying that drug clearance may be a more superior PK measure than drug concentration to predict outcomes and a better reflection of inflammatory burden than drug concentration. While highly correlated with one another, clearance performed better than drug concentration alone with respect to all investigated outcomes based on the higher AUC in the ROC curve analysis [50].

Regarding the association of vedolizumab clearance with clinical outcomes in patients with IBD, the ERELATE study showed that baseline vedolizumab clearance thresholds of <0.17 L/d and <0.16 L/d were associated with clinical and deep remission at week 52, respectively [51]. The same study showed that clearance in the lower quartiles was associated with higher rates of favorable therapeutic outcomes, including clinical and deep remission assessed either at week 14 or week 52 [51]. In a propensity-score-based case-matching analysis using data from the GEMINI 1, the RCT clinical response and remission rates at week 14 were 26.6% and 5.9%, respectively, in the highest vedolizumab clearance quartile (0.23 to <0.55 L/d) compared to 65.5% and 35.7, respectively, in the lowest vedolizumab clearance concentration quartile (0.03 to <0.14 L/d) at week 6 [36]. In a prospective multicenter study in children with IBD, starting with a vedolizumab baseline clearance of less than 0.161 L/d predicted a fecal calprotectin remission (<250 µg/g) at the end of the induction [40].

Currently, there are no data available regarding the association of ustekinumab, mirikzumab or risankizumab clearance with therapeutic outcomes in patients with IBD.

## 4. Clearance Monitoring and Model Informed Precision Dosing

The optimal dose of a biologic is not the same for every patient with IBD due to the high interindividual variability in the monoclonal antibodies’ PK, and one size does not fit all [3]. Clinical decisions based only on TDM are rather empirical as they are based on analog flowcharts or decision trees that refer more to a trial-and-error treatment optimization, underestimating the true value of TDM [53]. One of the most important aspects of PK is clearance. Clearance precedes changes in drug concentrations and can be an early predictor of disease relapse or development of immunogenicity. A recent study showed that a combination of infliximab concentration and clearance was a better predictor of CRP-based clinical remission compared to either one alone [54]. Another study showed that clearance may be even better than drug concentrations for predicting favorable therapeutic outcomes in patients with CD treated with adalimumab [50].

Baseline clearance, although imprecise as it is estimated only based on patients’ clinical and demographic data, can be used to identify patients at high risk of underexposure requiring early proactive TDM and an intensified induction regimen [55]. Drug clearance during biologic therapy is more accurately estimated as drug concentrations are taken into account and can be used by an MIPD tool towards a more personalized implementation of TDM, allowing patient-specific dosing forecasts to accurately achieve a predefined drug concentration target [55]. Of note, clearance monitoring and MIPD do not require TDM at a trough but can operate with intermediate drug concentrations. This allows for more flexibility in the sampling and an extended window of opportunity to adjust dosing. This is particularly important for reactive TDM, as patients are symptomatic and cannot wait until the next drug administration for a clinical decision to be made. A recent study showed an excellent correlation of forecasted infliximab trough concentrations from mid-cycle blood samples with measured trough specimens [56].

MIPD typically uses Bayesian modeling to estimate clearance based on population PK modeling and patient data and a software tool to predict the optimal dosing for achieving a target drug concentration. Preliminary data from retrospective and prospective studies both in adult and pediatric patients with IBD treated with infliximab support the concept of MIPD-based proactive TDM for maintaining therapeutic drug concentrations, showing the benefits of reduced immunogenicity, higher response rates, drug durability and fewer complications [52,57,58,59,60,61,62,63]. Most importantly, the PRECISION RCT (NCT02453776) demonstrated that a PK dashboard-based proactive TDM of infliximab was superior to standard dosing for sustaining remission during maintenance therapy [64].

The real-world impact of infliximab precision-guided dosing on management of patients with IBD was demonstrated by a recent study of 275 patients and 37 providers, where in 58% of cases, providers modified the treatment plans based on the results of the MIDP, including dose modifications (41%) and drug discontinuation (8%). Moreover, all providers reported that MIPD was beneficial in guiding treatment decisions and added more value to their practice than routine TDM [49]. A physiologically based pharmacokinetic model was recently used to predict the PK of anti-TNF agents in pregnant women, fetuses and infants to inform dosing decisions for infliximab, adalimumab and golimumab in pregnancy and vaccination regimens for infants [65]. However, wide utilization of MIPD in clinical practice is hindered by its limited availability, high cost, undetermined optimal TDM sampling based also on the assay used, the lack of clearly defined targets for drug concentrations among different IBD phenotypes and the complexity of bedside implementation. Preliminary data suggest that an MIPD tool can be embedded within the electronic health record, guiding clinical decisions in real time for pediatric patients with CD treated with infliximab or adalimumab [10,66].

## 5. Conclusions

Cumulative data suggest that clearance monitoring of biologics can predict therapeutic outcomes in IBD. Preliminary data also demonstrate the importance of clearance when estimated by MIPD tools for providing dosing recommendations towards treatment optimization. However, more prospective studies are needed to establish the role of MIPD of biologics in IBD and to investigate the efficacy of a novel therapeutic strategy that includes the combination of MIPD-based proactive TDM and pharmacodynamics monitoring. The ongoing RCTs TITRATE (NCT03937609), MODIFI (NCT04982172), REMODEL-CD (NCT05660746) and OPTIMIZE (NCT04835506) will shed more light on the role of MIPD of infliximab in IBD. Future perspectives regarding the use of MIPD include the incorporation of additional factors such as visceral adipose tissue, human leukocyte antigen haplotypes or drug concentration at the site of inflammation that could increase the accuracy of the estimated clearance and the dosing predictions.

## Figures and Tables

**Table 1:** Estimated clearance of biologics associated with therapeutic outcomes in patients with inflammatory bowel disease.

Author (Year)	Study Design	Drug	Population (N)	Estimated Clearance Time Point	Estimated Clearance Threshold, L/Day	Therapeutic Outcome (Time Point)
Battat (2021) [47]	Retrospective	IFX	ASUC (N = 39)	Baseline	≥0.627	Colectomy (6 months)
Vande Casteele (2019) [48]	ACT 1 and 2 RCTs	IFX	UC (N = 484)	Baseline	<0.397	MES ≤ 1 (week 8)
Vande Casteele (2019) [48]	ACT 1 and 2 RCTs	IFX	UC (N = 484)	Baseline	<0.364	MES ≤ 1 (week 30)
Peticollin (2019) [17]	Prospective	IFX	IBD (N = 91)	At time of de-escalation	>0.320	Relapse following treatment de-escalation
Whaley (2023) [21]	Prospective	IFX	ASUC * (N = 38)	Day 3 after drug initiation	>0.480	Colectomy
Chung (2023) [24]	Retrospective	IFX	CD (N = 85)	Baseline	<0.230	Remission (5 months)
Chung (2023) [24]	Retrospective	IFX	CD (N = 85)	Baseline	<0.238	Remission (10 months)
Chung (2023) [24]	Retrospective	IFX	CD (N = 85)	Baseline	<0.243	Remission (16 months)
Chung (2023) [24]	Retrospective	IFX	CD (N = 85)	End of induction	<0.230	Remission (5 months)
Chung (2023) [24]	Retrospective	IFX	CD (N = 85)	End of induction	<0.213	Remission (10 months)
Chung (2023) [24]	Retrospective	IFX	CD (N = 85)	End of induction	<0.252	Remission (16 months)
Vermeire (2022) [22]	Prospective	IFX	IBD (N = 276)	Baseline, weeks 2, 6, 14	<0.250	CRP-based clinical remission
Abraham (2023) [49]	Prospective	IFX	IBD (N = 275)	Maintenance	>0.294	Active disease, drug discontinuation
Wright (2023) [50]	Retrospective	ADM	CD (N = 237)	Maintenance ^#^	<0.350	SES-CD < 3
Wright (2023) [50]	Retrospective	ADM	CD (N = 237)	Maintenance ^#^	<0.280	FC < 100 ug/g
Wright (2023) [50]	Retrospective	ADM	CD (N = 237)	Maintenance ^#^	<0.300	CRP-based clinical remission
Lefevre (2022) [32]	PRECiSE 1 and 2 RCTs	CZP	CD (N = 964)	Baseline	>0.500	Drug TC < 36 μg/mL (week 6)
Vande Casteele (2022) [51]	Retrospective	VDZ	IBD (N = 695)	Baseline	<0.170	Clinical remission (week 52)
Vande Casteele (2022) [51]	Retrospective	VDZ	IBD (N = 695)	Baseline	<0.160	Deep remission (week 52)
Osterman (2019) [36]	GEMINI 1 RCT	VDZ	UC (N = 693)	Week 6	<0.140	Clinical response (week 14)
Colman (2022) [40]	Prospective	VDZ	IBD (N = 21)	Baseline	<0.161	FC < 250 mg/g (week 14)

* Pediatric; ^#^ The sampling time for adalimumab pharmacokinetics were matched to the study visit assessment, occurring anytime relative to the last dose. IFX: infliximab; ADM: adalimumab; CZP: certolizumab pegol; VDZ: vedolizumab; ASUC: acute severe ulcerative colitis; IBD: inflammatory bowel disease; MES: Mayo endoscopic score; CD: Crohn’s disease; UC: ulcerative colitis; FC: fecal calprotectin; CRP: C-reactive protein; SES-CD: Simple Endoscopic Score for Crohn’s Disease; RCT: randomized controlled trial; TC: trough concentration.

## Data Availability

Not applicable.
